# Stressful Life Events in Different Social Contexts Are Associated With Self-Injury From Early Adolescence to Early Adulthood

**DOI:** 10.3389/fpsyt.2020.487200

**Published:** 2020-10-27

**Authors:** Annekatrin Steinhoff, Laura Bechtiger, Denis Ribeaud, Manuel Eisner, Lilly Shanahan

**Affiliations:** ^1^Jacobs Center for Productive Youth Development, University of Zurich, Zurich, Switzerland; ^2^Institute of Criminology, University of Cambridge, Cambridge, United Kingdom; ^3^Department of Psychology, University of Zurich, Zurich, Switzerland

**Keywords:** non-suicidal self-injury, NSSI, self-harm, adolescence, stress, life events, sex differences

## Abstract

Self-injury often arises as a maladaptive coping strategy used to alleviate distress. Past research has typically examined how chronic stressors in a specific context are associated with self-injury. Little is known about the unique and cumulative associations between acute stressful life events that occur in different social contexts and self-injury among adolescents. This is especially the case for males, for whom the etiology of self-injury is understudied. We examine the unique and cumulative contributions of stressful life events in the contexts of adolescents' school life, peer networks, intimate relationships, and family life to self-injurious behavior in males and females from the community. Our data comes from a prospective-longitudinal community-representative study, the Zurich Project on the Social Development from Childhood to Adulthood (*z-proso*). Our sample consists of 1,482 adolescents (52% male) assessed at ages 13, 15, 17, and 20. At each age, adolescents reported whether they had engaged in self-injury during the previous month. They also reported stressful life events in the school, peer, intimate relationships, and family contexts, typically since the last assessment. Stressful life events in the peer context were consistently associated with self-injury. In the contexts of school, intimate relationships, and family, some associations were age- or sex-specific. For example, mid-adolescent females were more likely than mid-adolescent males to use self-injury when faced with stressful events in school and intimate relationships. With respect to risk accumulation, females' risk of self-injury increased with each additional life event between the ages of 13 and 17, beginning at 2+ events. This pattern did not hold for males. In early adulthood, 4+ life events were associated with an increased risk of self-injury, which suggests that the thresholds for the number of life events needed to trigger self-injury increased from adolescence to young adulthood. Our findings suggest that reducing risk of stressful events in different social contexts, and improving young people's coping skills could help reduce their risk of self-injury. New or revised theoretical models may be needed to better understand the emergence of self-injury in males.

## Introduction

Adolescent self-harm, including self-injury, is a serious public health issue across the globe (1, 2). In recent years, several theories have emerged to explain why adolescents engage in this behavior (3, 4). One influential theory maintains that self-injury is a maladaptive coping or affect-regulation strategy that some adolescents use when they feel overwhelmed or over-aroused by emotional distress or unmanageable social demands (3).

Despite this progress in theory development, the circumstances under which self-injury arises are not well-characterized (5). Here we aim to answer three research questions: First, is adolescent self-injury more likely to occur in response to stressful life events in certain social contexts rather than others (e.g., peer networks vs. family life)? Second, is there a particular number (or threshold) of stressful life events that causes stress to become so overwhelming that adolescents engage in self-injury? Third, do associations between stressful life events and self-injury differ between males and females? Answering these questions may contribute to a more nuanced understanding of the etiology of self-injury and provide insights into when, for whom, and by what means the risk of self-injury could be reduced.

Adolescence is a time of increased interpersonal sensitivity and interpersonal stress (6–8). While confronting the psychosocial strains associated with the social transitions of adolescence, young people simultaneously undergo biological transitions that can compound their levels of and reactions to stress (9, 10). For example, in addition to pubertal changes, adolescents' stress responses are fundamentally reshaped during early and mid-adolescence. Therefore, stress reactivity may temporarily increase, meaning that a stressor that would be well-tolerated by a person during other developmental stages could be experienced as overwhelming during adolescence (10). Here we considered the potential age-specific associations between stressful life events and self-injury by using four repeated assessments between the ages of 13 and 20 years.

Social distress can occur in all of the main social contexts of adolescence, including in school life, peer networks, intimate relationships (e.g., with best friends or romantic partners) and family contexts. Prior research has mostly focused on associations between chronic stress (i.e., stress that persists over prolonged periods) in particular social contexts and self-injury. Compared to such chronic adversities, stressful life events are discrete and short-lived but, nevertheless, result in increased stress levels and potential psychological problems (11, 12), which may include self-injury. The body of work on life events and adolescent self-injury is relatively small (13–16) and one limitation is that most previous studies have not examined whether stressful life events in some contexts (e.g., peers) are more strongly associated with self-injury than events in other contexts (e.g., family). Such a direct comparison can only be done when events in several contexts are included in one and the same study. Therefore, the first aim of our study is to examine associations between stressful events in different social contexts and self-injury from early adolescence to early adulthood.

In the school context, for example, adolescents can be exposed to considerable pressures as they are expected to strive for academic and future professional success and must undergo several educational transitions. School pressures and anxiety associated with school performance are stressful and could lead to adolescents engaging in self-injury as a coping strategy (17). School stressors are also associated with low self-esteem (18), which is, in turn, associated with self-injury (19). However, the role of acute stressful events, such as school failures (e.g., grade retention, failing exams), on adolescent self-injury has not been extensively investigated.

Adolescents also often face stressful life events as they undergo transitions in their peer networks (peer context), form intimate bonds with best friends and romantic partners (intimate relationships context), and re-negotiate their roles in relation to parents and siblings (family context) (9, 20). Evidence of associations between chronic adversity relating to peer and romantic relationships and self-injury is consistent (13, 19, 21, 22), whereas evidence of associations between family-related adversity and self-injury is mixed (21, 23, 24). Evidence of the unique role of acute life events in these contexts (e.g., being physically attacked by peers, the breakup of intimate relationships, or experiences of loss in the family context) is largely missing from the literature.

The second aim of our study is to address the question of what happens when stressful life events accumulate over a short period of time, possibly consuming adolescents' lives and coping resources. It is plausible that one or two life events could be compensated for (or buffered) by relying on protective factors, such as constructive coping strategies or social support. However, multiple life events over a short period of time could contribute to overwhelming stress that triggers self-injury, irrespective of the social context of these events. Evidence of the detrimental effects of risk accumulation can be found in cumulative risk research, which shows that the risk of negative outcomes, including psychological problems, increases with each additional stressor (25). Research on adverse childhood experiences (ACEs) also shows graded associations between the number of exposures and outcomes. Notably, this work has reported that those with four or more ACE categories are at particular risk (26). Recent research has also shown that higher numbers of life events are associated with an increased risk of self-injury (13–16). However, thresholds of stressful life events associated with particularly dramatic increases in the risk of self-injury, comparable to those in studies of ACEs, have not yet been investigated. Therefore, this study examines whether an accumulation of recent life events is associated with self-injury and how many events cause this association to become particularly strong.

Much of the existing research has been unable to examine whether associations between stressful life events and self-injury change with age because most studies are cross-sectional or short-term longitudinal in design. Given the many changes that occur during adolescence, including changes in social priorities and stress reactivity (9, 10), it seems plausible that the strength of the association between life events and self-injury could change with age. For example, school-related failures may become increasingly stressful in late adolescence when important educational and professional transitions are pending, whereas other events, such as severe forms of peer victimization, may be highly relevant to well-being throughout adolescence (24, 27). Romantic break-ups could be particularly stressful in cases of an adolescent's first serious relationship with an intimate romantic partner (e.g., in mid- or late adolescence). Indeed, one study found an association between romantic stress and increased risk of self-injury, but only in girls during advanced puberty (21). Finally, family-related experiences of loss are known to increase young people's risk of mental health problems (28), but family-related life events encountered during early adolescence may be more critical than those in later adolescence, when young people become increasingly independent from the family.

Another caveat of previous research is that many studies use predominantly female or clinic-recruited samples (29). Therefore, associations between life events and self-injury among adolescents from the community are not well-documented. In addition, male self-injury and its etiology are poorly understood. It is possible that the contexts and numbers of life events that are followed by self-injury differ between males and females (30). A recent study conducted on the same sample used in this work showed that the reasons that males with self-injury reported for use of mental health services tended to differ from those of females with self-injury (31). This also suggests that the triggers of self-injury could differ by sex. It is also possible that males do not necessarily engage in self-injury when dealing with stressors but use other (maladaptive) coping strategies instead (e.g., substance use, aggressive behaviors). Therefore, the third aim of our study is to examine sex differences across all associations between life events and self-injury.

## Methods

### Sample and Procedures

Our data is taken from four waves of the ongoing longitudinal Zurich Project on the Social Development from Childhood to Adulthood [*z-proso*; (32, 33)]. Participants were selected using a cluster-stratified randomized sampling approach. In 2004, a sample of 1,675 children from 56 primary schools was randomly selected from 90 public schools in the city Zurich, Switzerland's largest city. Stratification was performed taking into account school sizes and socioeconomic background of the school districts. The sample was largely representative of first-graders attending public school in the city of Zurich. Participants were followed until 2018, when they were 20 years old.

The current study uses data that was mainly collected from participants aged 13 onward, when self-injury was first assessed and when adolescents face many stressors and transitions [*N* = 1,362; *N* = 1,443; *N* = 1,305; *N* = 1,180 at mean ages 13 (grade 7), 15 (grade 9), 17 (grade 11), and 20, respectively]. For example, in Zurich, adolescents are academically tracked into vocational school and college-bound tracks based on their academic performance at ages 12/13 and 15/16. In the course of these tracking decisions, many adolescents take high-stakes tests and must make other important educational decisions.

Of those who participated at least once between ages of 13 and 20 (*N* = 1,482), 52% were male. Consistent with Switzerland's immigration policies and the city's diverse population, participants had parents who had been born in over 80 different countries, and 76% of the adolescents had grown up with at least one parent with an immigration background. The majority of adolescents were born in Switzerland (91%). The parental educational background of participants was diverse; in 26% of households, at least one parent held a university degree. The mean household occupational status, measured using the International Socio-Economic Index of Occupational Status (34), was 45.74 (SD = 19.24). This internationally comparable index of socio-economic status is based on occupation-specific income and the required educational level, with scores ranging from 16 (e.g., unskilled worker) to 90 (e.g., judge) in our sample.

The study is consistent with national and international ethical standards and was approved by the responsible ethics committee. Adolescents provided written consent for their study participation, and parents of those aged 15 and younger could choose not to have their child participate in the study. Data were collected from groups of 5–25 participants in classroom settings with paper-and-pencil questionnaires up to age 17 and in a computer laboratory setting with computer-administered surveys at age 20. Completing the surveys typically took ~90 min. Adolescents received a cash incentive for their participation, which increased from ~$30 at age 13 to $75 at age 20.

### Measures

Self-injury was self-reported at ages 13, 15, 17, and 20 years. Respondents were asked how often they had harmed themselves on purpose during the previous month. Several example behaviors were provided (i.e., “cut my arm,” “tore open wounds,” “hit my head,” “tore out my hair”). Respondents were not asked whether self-injury was pursued with suicidal intent; therefore, our assessment does not distinguish between suicidal and non-suicidal self-injury. However, the example behaviors provided to participants were prototypical non-suicidal self-injury behaviors. Answers were recorded on a five-point scale (1 = “never,” 2 = “rarely,” 3 = “sometimes,” 4 = “often,” and 5 = “very often”). For our analyses, we use a dichotomized variable (0 = “never,” 1 = “at least rarely,” with the latter implying at least once during the previous month). Twenty-seven percent of those who participated at all assessments between ages 13 and 20 reported self-injury at some point during adolescence [for more detail on the development of self-injury among males and females in this sample, see (31)].

**Figure 1:**
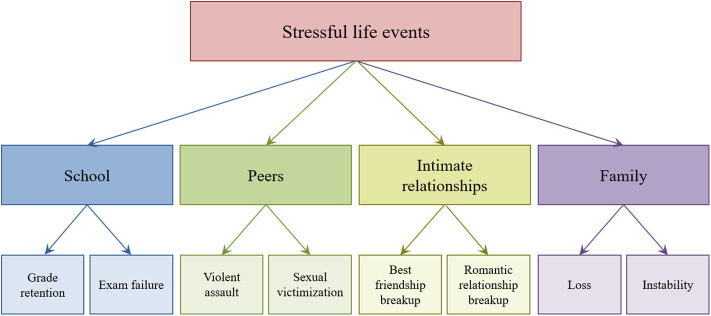
Eight stressful events in four social contexts of adolescents' everyday lives.

Stressful life events in the four main adolescent social contexts (i.e., school, peer networks, intimate relationships, and family) were also reported at ages 13, 15, 17, and 20. The adolescents were presented with a list of events and asked to indicate whether an event had occurred during the past two years (at ages 13–17) or three years (at age 20). These recall time frames allowed us to assess all stressful events since the previous interview. The only exception were events in the peer context, which were assessed in a separate section of the questionnaire, which assessed events in the previous year only. Within each of the four contexts, two types of stressful events were captured (see Figure 1). School-related events were (1) failure in an important exam and (2) grade retention. For stressful life events involving peer networks, we focused tightly on peer violence because of our focus on short-lived life events. Other forms of peer victimization (e.g., bullying) are often chronic rather than short-lived, and, thus, do not capture discrete acute experiences. Events involving peer violence were defined as (1) any violent assault by a peer (with or without a weapon) and (2) sexual victimization (i.e., sexual assault or sexual harassment by a peer). At ages 17 and 20, the wording of these items changed from “peers” to “others,” [but most violent or sexual assaults at these ages are likely to be committed by peers (35)]. Stressful events in the context of intimate relationships were identified as (1) the breakup of a romantic relationship and (2) the breakup of a best friendship. Family events were (1) loss of a family member (i.e., death of a parent or sibling) and 2) exposure to family instability (i.e., parental separation, a parent's new partner moving into the household, parental job loss, or parental hospitalization). Although *z-proso* assessed a variety of other life events, we limit our analyses to events that were measured at every assessment included here.

In the current paper, we use (a) binary assessments, indicating whether any event had occurred in a particular social context (0 = no event, 1 = at least one event), and (b) a cumulative sum score, indicating how many events had occurred at least once (possible range = 0–8). This cumulative score represents the variety of events that had occurred. We also created (c) a cumulative sum score indicating the number of social contexts within which any event had occurred (possible range = 0–4).

Control variables were chosen to address potential pre-existing differences in life circumstances that may have affected the probability of stressful events and self-injury. The control variables were (i) high parental education (1 = at least one parent with a tertiary education degree, 0 = both parents with some lower educational level), (ii) child's educational level at the previous assessment [1 = academic high school (called “high” in our tables) vs. 0 = other at ages 13, 15, and 17], (iii) parental separation/divorce by child age 11, which was included in order to adjust for pre-existing family stressors or instability, and (iv) migration background (1 = both parents born abroad vs. 0 = at least one parent born in Switzerland).

### Analytic Strategy

To answer our three research questions, regression models were specified in M*Plus* V7 (36) using the maximum likelihood robust (MLR) estimator, which is a useful estimator for categorical outcomes and provides logit coefficients and odds ratios (ORs) with confidence intervals (i.e., for logistic regression models).

The context-specific relevance of stressful events for adolescent self-injury (first research question) was analyzed in two steps. First, four separate models were specified for each of the four social contexts to test associations between the occurrence of a stressful event in a specific context and self-injury at each age. Second, all four variables that represent the occurrence of a stressful life event in each of the four contexts were included within a single model to test whether events in particular contexts were unique risk factors for self-injury. All associations were adjusted for sex, parental educational background, adolescent educational level at the previous assessment, parental separation/divorce by age 11, and migration background.

When examining life event accumulation and self-injury at a given age (second research question), we specified models in which the number of events reported at that assessment was included, first, as a continuous variable ranging from 0–8 (cumulative model) and, second, as a categorical variable indicating the occurrence of 0, 1, 2, 3, or 4+ events (threshold model). In the threshold model, zero events was used as the reference category and the other categories were included as dummy variables. In an additional similar set of analyses, we compared the associations between self-injury and the number of contexts, in which any event had occurred (0, 1, 2, or 3+ contexts).

To assess sex-specific associations (third research question), we ran separate analyses for males and females and also included interaction terms (sex^*^context of event or sex^*^number of events) in the overall models. All possible interaction terms (sex^*^context of event or sex^*^number of events) were included separately (i.e., one at a time).

In a set of follow-up analyses, we extended the main models and also included auto-regressive paths between self-injury at two consecutive assessments, thus controlling for a potential overlap between life events and prior self-injury.

A small percentage of attrition occurred at each assessment wave and potential bias could have arisen from selective attrition mechanisms (33, 37, 38). To avoid such bias, we used model-based multiple imputation (MI). This procedure takes into account the uncertainty associated with imputing data. MI is considered a gold standard for handling missing data in developmental research (39). We specified our models within a structural equation modeling framework, which allowed us to estimate associations between life events and self-injury at the four assessments in the same statistical model (i.e., for each set of predictor variables [events in a particular context, events in all contexts, or accumulated number of events], we estimated four regressions simultaneously, for the self-injury outcomes at ages 13, 15, 17, and 20). With this technique, all adolescents who participated in the study at least once between the ages of 13 and 20 could be included in our imputation models (*N* = 1,482; 767 males and 715 females); thereby maximizing the accuracy of our estimates. Specifically, twenty imputed data sets were generated, and the estimates reported here were averaged across the complete data sets.

## Results

### Descriptive Statistics

In a previous paper based on the same sample, we reported that self-injury prevalence was highest in early adolescence and then decreased in the overall sample and in males but peaked in mid-adolescence among females (31). In the current study, the numbers of stressful life events adolescents reported were M(SD) = 1.47(1.27), 1.33(1.21), 1.20(1.10), 1.96(1.51) at ages 13, 15, 17, and 20, respectively. An overview of the average number of events, the frequency of specific types of events, and the contexts in which stressful events occurred among youth with and without self-injury are provided in Supplementary Section 1. Overall, the burden from stressful life events was considerably higher among those with self-injury than among those without. The occurrence of life events in some contexts was associated with the occurrence of life events in other contexts, but the sizes of most associations were rather low [OR ranging from 1.06 (not significant) to 2.99; see Supplementary Section 2 for all associations]. The strongest associations were observed between peer violence and intimate relationship breakups.

### Contexts of Stressful Life Events and Self-Injury

#### Overall Sample

**Figure 2:**
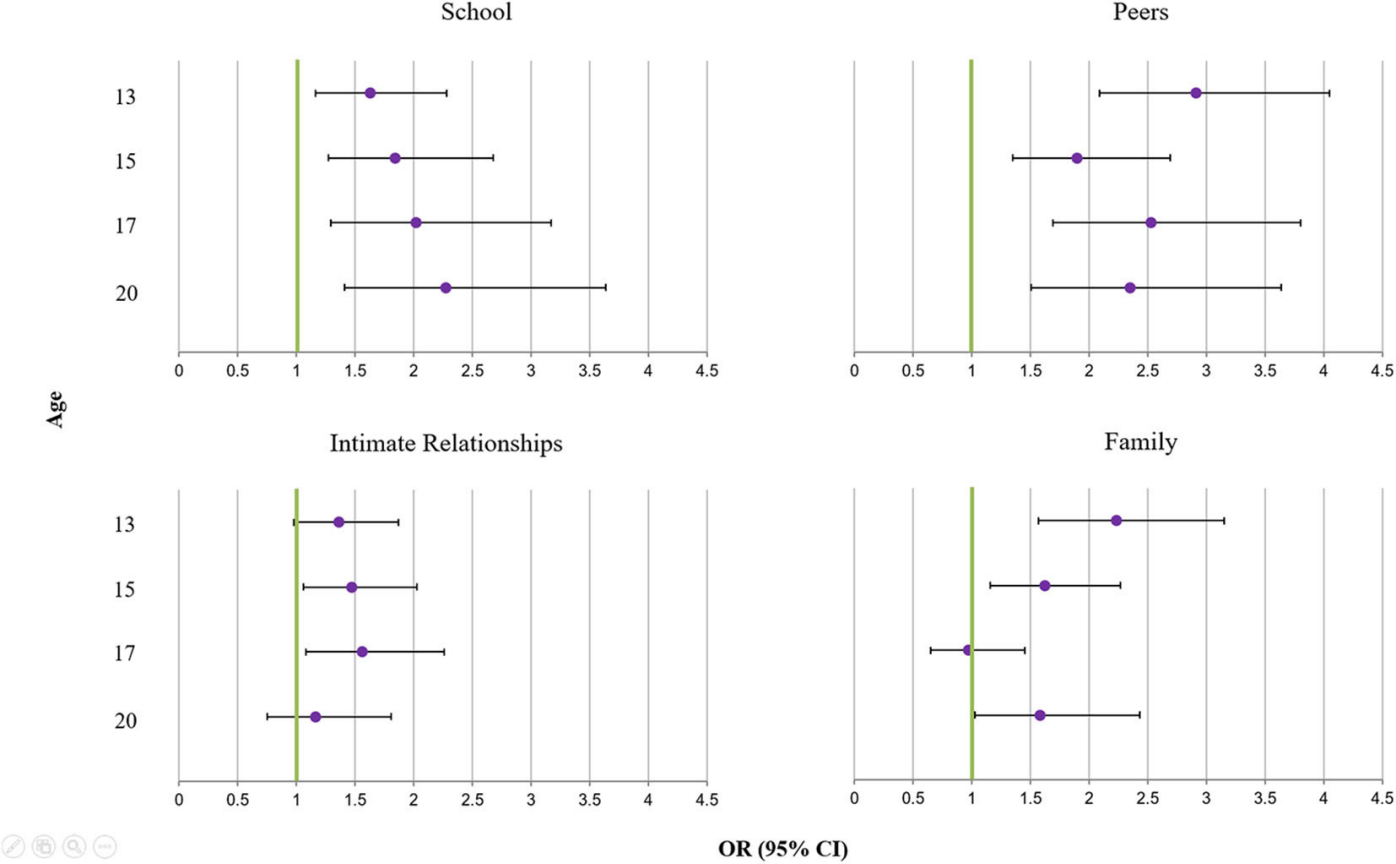
Associations between life events in four social contexts (separate models) and self-injury: odds ratios and 95% confidence intervals from logistic regressions, adjusted for sex, parental educational background, adolescent educational level at previous assessment, parental divorce by child age 11, and migration background.

The separate models showing associations between self-injury and events in single specific contexts (Figure 2) revealed that school-related events were associated with an increased risk of self-injury at all ages (*b* = 0.49–0.82, *p* = 0.001–0.004). Confidence intervals for the ORs overlapped across the different ages. Nevertheless, the sizes of the associations between school events and self-injury appeared to increase with age. Life events involving peer violence were consistently associated with self-injury from early adolescence until early adulthood. Except at age 15, the effect size was OR > 2 (*b* = 0.64–1.07, *p* < 0.001 at all time-points), suggesting a sizeable association between violent peer experiences and self-injury. Life events in the context of intimate relationships were also associated with self-injury but with comparably smaller effect sizes (see Figure 2) and higher *p*-values (*b* = 0.30–0.45, *p* = 0.018–0.066 between age 13 and 17); the association was non-significant at age 20. The confidence intervals for the ORs of the intimate relationships associations were considerably smaller compared to most other events, which likely reflects the fact that a larger group of adolescents had experienced life events in this context compared to other contexts.

Family-related life events were associated with self-injury at ages 13 and 15 (*b* = 0.48–0.80, *p* < 0.001–0.005), but not at age 17. At age 20, the association was significant again (*b* = 0.46, *p* = 0.036). Specifically, an initial decrease of effect sizes for family-related events from ages 13 to 17 was followed by an increase at age 20 (see Figure 2).

The results of the models showing associations between self-injury and stressful events in any of the four social contexts show that most associations observed in the separate models are unique and remain significant, at least at the statistical trend level (i.e., *p* < 0.10; see Table 1). Life events involving peer violence and school events emerged as strong correlates of self-injury at all ages. Associations between stressful life events in the context of intimate relationships and subsequent self-injury were consistently weak and mostly non-significant.

**Table 1:** Associations between self-injury between age 13 and 20 (dependent variables) and life events: results from multivariate logistic regressions with multiple imputation (*N* = 1,482).

				**95% CI**	
	**Logit coeff. *b***	***p***	**OR**	**Lower**	**Upper**
**Self-injury age 13**					
Sex (male)	−0.07	0.686	0.93	0.67	1.30
High parental education	−0.24	0.324	0.79	0.49	1.26
Parents divorced by child age 11	0.36	0.082	1.43	0.96	2.14
Migration background	−0.05	0.764	0.95	0.67	1.34
*Context of event*					
School	0.37	0.039	1.44	1.02	2.05
Peers	1.00	<0.001	2.71	1.93	3.81
Intimate relationships	−0.03	0.846	0.97	0.69	1.35
Family	0.65	<0.001	1.92	1.37	2.70
**Self-injury age 15**					
Sex (male)	−0.79	<0.001	0.45	0.32	0.65
High parental education	−0.47	0.056	0.63	0.39	1.01
Parents divorced by child age 11	0.17	0.447	1.18	0.77	1.81
Migration background	−0.26	0.142	0.77	0.54	1.09
Child education level (high)^a^	−0.11	0.652	0.89	0.55	1.46
*Context of event*					
School	0.51	0.009	1.66	1.13	2.44
Peers	0.54	0.003	1.72	1.21	2.46
Intimate relationships	0.27	0.121	1.31	0.93	1.83
Family	0.42	0.015	1.52	1.08	2.14
**Self-injury age 17**					
Sex (male)	−0.76	0.001	0.47	0.31	0.72
High parental education	−0.32	0.255	0.73	0.42	1.26
Parents divorced by child age 11	0.15	0.546	1.16	0.72	1.85
Migration background	−0.25	0.242	0.78	0.52	1.18
Child education level (high)^a^	0.08	0.750	1.08	0.67	1.75
*Context of event*					
School	0.61	0.013	1.84	1.14	2.99
Peers	0.81	<0.001	2.24	1.49	3.38
Intimate relationships	0.35	0.083	1.41	0.96	2.09
Family	−0.05	0.828	0.96	0.63	1.44
**Self-injury age 20**					
Sex (male)	−0.58	0.017	0.56	0.35	0.90
High parental education	0.14	0.651	1.15	0.64	2.06
Parents divorced by child age 11	0.29	0.291	1.33	0.78	2.26
Migration background	0.04	0.877	1.04	0.67	1.61
Child education level (high)^a^	−0.34	0.212	0.71	0.42	1.21
*Context of event*					
School	0.80	0.001	2.24	1.41	3.53
Peers	0.76	0.001	2.15	1.34	3.44
Intimate relationships	−0.07	0.788	0.94	0.59	1.50
Family	0.41	0.073	1.50	0.96	2.34

a *Education level at the previous assessment*.

#### By Sex

**Figure 3:**
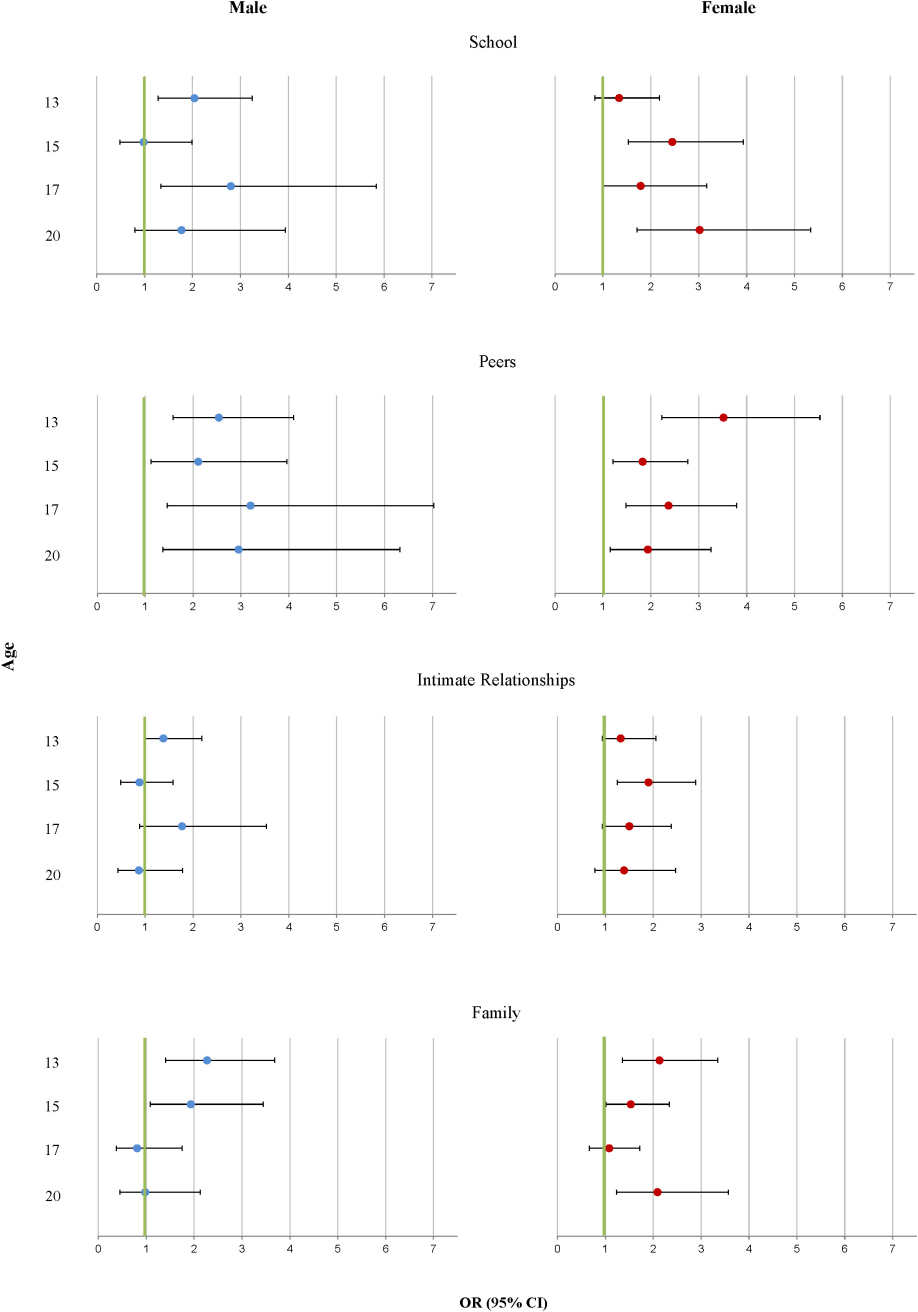
Sex-specific associations between stressful life events in four social contexts (separate models) and self-injury: odds ratios and 95% confidence intervals from logistic regressions, adjusted for parental educational background, adolescent educational level at previous assessment, parental divorce by child age 11, and migration background.

The results from the models that concerned events in a single context revealed some sex-specific patterns (Figure 3). With regard to school-related events, a significant sex interaction emerged at age 15 (*p* = 0.045), showing that school-related life events at age 15 were associated with increased risk of self-injury only in females (*b* = 0.90, *p* < 0.001), and not in males. Life events involving peer violence were consistently associated with an increased likelihood of self-injury among both males and females, there were no significant sex interactions. With regard to intimate relationship breakups, the sex difference was significant at age 15 (*p* = 0.032). At that age, intimate relationship breakups were associated with an increased risk of self-injury among females only (*b* = 0.64, *p* = 0.003). For family-related events, no significant sex interaction emerged.

The findings from the separate models were mostly replicated when events in the four social contexts were included in a single model, except that intimate relationships breakups were no longer associated with self-injury in females at age 17 (see Supplementary Section 3 for sex-specific multivariate models including any event in all four contexts). *p*-values of the interaction terms sex^*^context of event in the final overall model were 0.045 in the case of school-related events and 0.051 in the case of intimate relationship breakups at age 15.

#### Summary

A summary shows that school-related stressful life events and peer violence were consistently associated with self-injury across all ages. Intimate relationship breakups were also associated with self-injury, but only sporadically and with a smaller effect size. Family-related life events were associated with self-injury particularly from early to mid-adolescence (ages 13 and 15) and, with a weaker association, at age 20. Significant sex differences emerged in the contexts of school and intimate relationships at age 15. Specifically, mid-adolescent females were more likely than males to engage in self-injury when faced with stressful events in these contexts.

### Accumulation of Stressful Life Events and Self-Injury

#### Overall Sample

**Figure 4:**
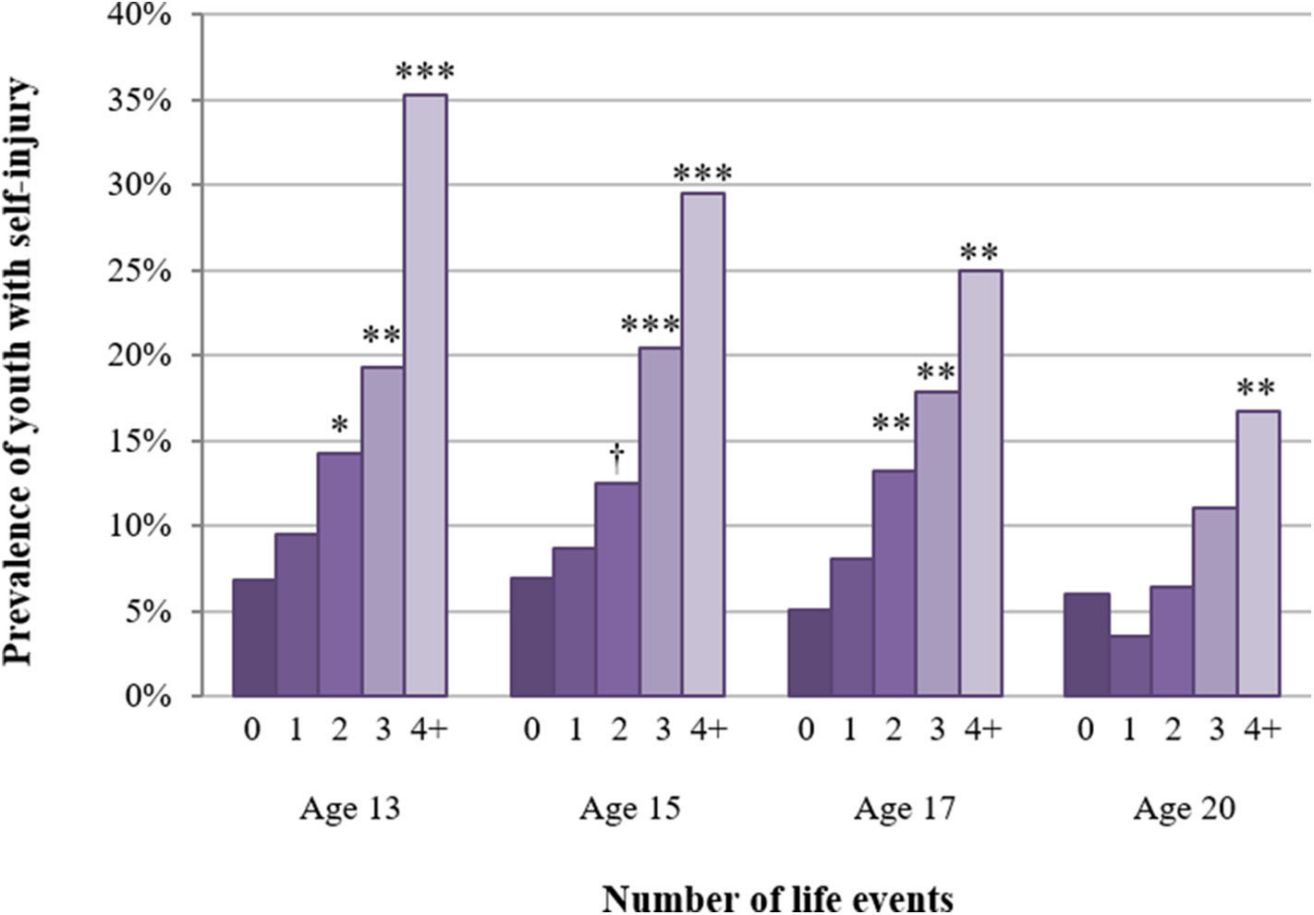
Overall threshold model: proportion of youth with self-injury among those with zero to four or more stressful life events from ages 13 to 20. Asterisks represent *p*-value of the contrast between a particular number of events vs. zero events (reference) from a model that adjusted for sex, parental educational background, adolescent educational level at previous assessment, parental divorce by child age 11, and migration background. †*p* < 0.10; *p < 0.05, **p < 0.01, ***p < 0.001.

The model with an accumulated score of stressful life events (continuous variable) showed that, across adolescence, a greater number of life events was associated with an increased risk of self-injury (OR = 1.58, 95% CI = 1.38–1.81, *b* = 0.46, *p* < 0.001; OR = 1.46, 95% CI = 1.27–1.68, *b* = 0.38, *p* < 0.001; OR = 1.52, 95% CI = 1.28–1.80; *b* = 0.42, *p* < 0.001; OR = 1.42, 95% CI = 1.19–1.68, *b* = 0.35, *p* < 0.001 at ages 13, 15, 17, and 20, respectively). The model using the number of events as a categorical variable (threshold model) showed that rates of self-injury were lowest among youth who had experienced zero or one stressful event (Figure 4). Beginning at two life events, however, rates of self-injury increased with an increasing number of events from ages 13 to 17 (Table 2). At age 20, only the contrast between four or more events and zero events was significant (Supplementary Section 4 shows the distribution of the accumulated life events variable used in the thresholds model).

**Table 2:** Threshold models: contrast between zero (reference category) and other numbers of stressful life events associated with self-injury, adjusted for sex, parental educational background, adolescent educational level at previous assessment, parental divorce until age 11, and migration background.

**Age**	**No. of event types**	**Overall**				**Male**				**Female**			
		**Logit coeff. *b***	***p***	**OR**	**95% CI**	**Logit coeff. *b***	***p***	**OR**	**95% CI**	**Logit coeff. *b***	***p***	**OR**	**95% CI**
13 years	1	0.24	0.350	1.27	0.77–2.11	0.11	0.749	1.12	0.56–2.23	0.35	0.381	1.42	0.65–3.08
	2	0.66	0.011	1.93	1.16–3.19	0.34	0.359	1.41	0.68–2.91	0.91	0.016	2.49	1.19–5.23
	3	0.95	0.001	2.59	1.50–4.47	0.21	0.647	1.23	0.50–3.02	1.45	<0.001	4.27	1.97–9.24
	4+	1.79	<0.001	6.00	3.38–10.65	1.88	<0.001	6.56	3.02–14.25	1.69	<0.001	5.40	2.23–13.07
15 years	1	0.14	0.584	1.15	0.70–1.91	−0.40	0.284	0.67	0.32–1.39	0.67	0.076	1.95	0.93–4.06
	2	0.45	0.088	1.58	0.94–2.65	0.36	0.348	1.43	0.68–3.02	0.75	0.049	2.12	1.00–4.49
	3	0.98	<0.001	2.65	1.55–4.54	−0.06	0.923	0.95	0.31–2.91	1.54	<0.001	4.64	2.19–9.85
	4+	1.50	<0.001	4.49	2.35–8.59	0.91	0.121	2.49	0.79–7.86	1.97	<0.001	7.20	3.02–17.20
17 years	1	0.25	0.377	1.28	0.74–2.21	0.12	0.780	1.13	0.48–2.65	0.24	0.510	1.27	0.62–2.59
	2	0.78	0.007	2.19	1.24–3.85	0.73	0.118	2.08	0.83–5.21	0.75	0.034	2.12	1.06–4.23
	3	1.12	0.001	3.07	1.62–5.80	0.86	0.164	2.37	0.70–7.98	1.12	0.005	3.06	1.41–6.65
	4+	1.36	0.002	3.88	1.63–9.20	1.89	0.014	6.63	1.47–29.83	1.09	0.038	2.97	1.06–8.32
20 years	1	−0.64	0.103	0.53	0.25–1.14	−0.54	0.361	0.59	0.19–1.85	−0.64	0.257	0.53	0.18–1.60
	2	−0.04	0.907	0.96	0.48–1.93	−0.52	0.351	0.60	0.20–1.77	0.41	0.420	1.51	0.56–4.10
	3	0.48	0.163	1.62	0.82–3.20	0.67	0.264	1.95	0.60–6.32	0.51	0.298	1.66	0.64–4.33
	4+	0.92	0.004	2.51	1.35–4.67	0.65	0.249	1.91	0.64–5.75	1.22	0.012	3.40	1.31–8.79

*Results from logistic regressions with multiple imputation [N (overall) = 1,482, n (male) = 767, n (female) = 715]*.

#### By Sex

**Figure 5:**
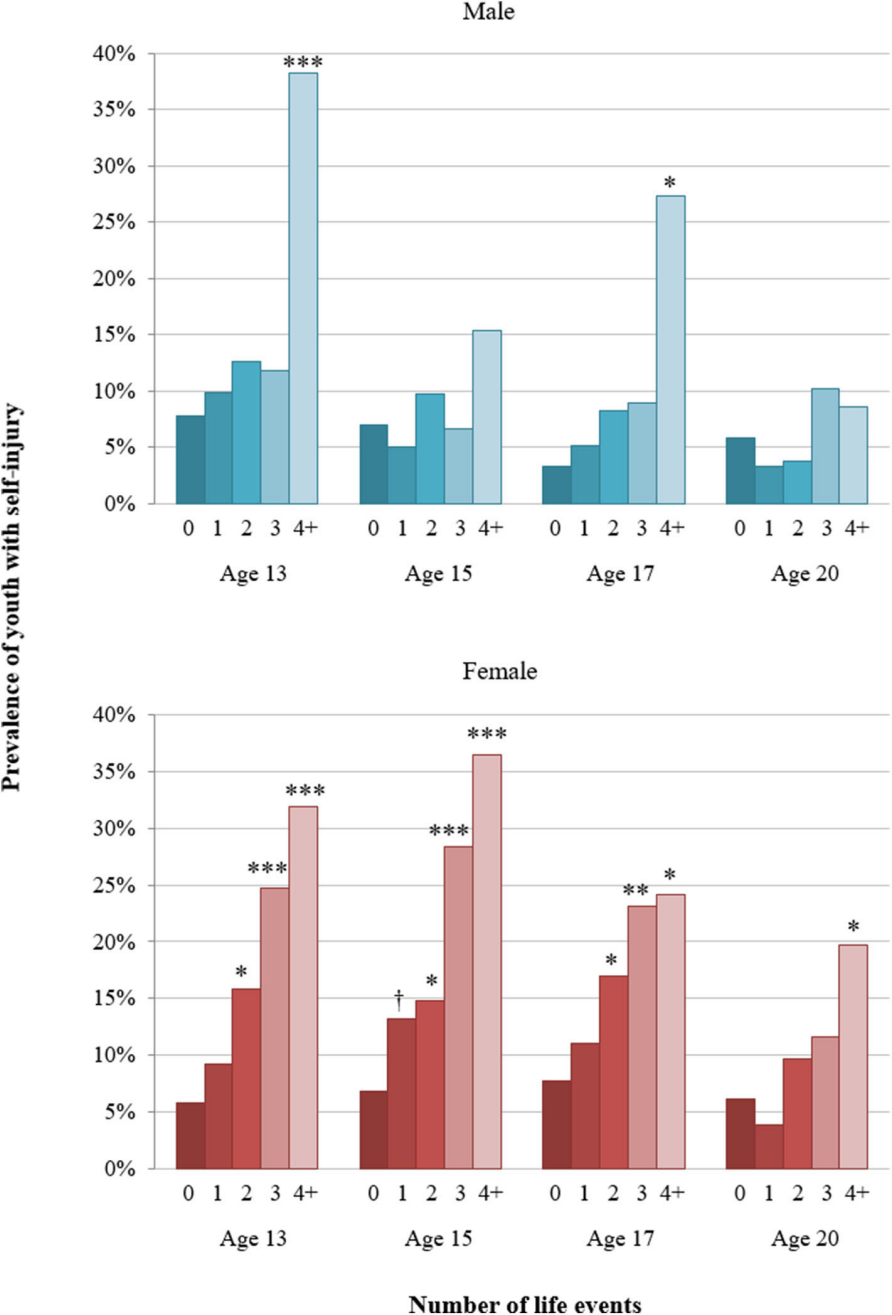
Sex-specific threshold models: proportion of youth with self-injury among those with zero to four or more stressful events from ages 13 to 20. Asterisks represent *p*-value of the contrast between particular numbers of life events vs. zero events (reference) from models that adjusted for parental educational background, adolescent educational level at previous assessment, parental divorce by child age 11, and migration background. †*p* < 0.10; *p < 0.05, **p < 0.01, ***p < 0.001.

The cumulative models indicated that associations between the number of stressful events and self-injury partially differed for males and females; there was a significant interaction of sex^*^number of events at age 15 (*p* = 0.046). The threshold models showed that in males, significant associations were observed at ages 13 and 17, when exposure to four or more life events was associated with an increased risk of self-injury (Figure 5; Table 2). In females, the threshold for increased risk of self-injury was two or more events between ages 13 and 17 and four or more events in early adulthood (age 20).

#### Accumulation of Stressful Life Events Across Contexts

An additional similar set of analyses was conducted to examine whether the number of contexts in which stressful events had occurred was associated with self-injury (see Supplementary Section 5). The rationale here was to test the hypothesis that when more than one context of adolescents' lives is affected by stressful life events, the risk of self-injury may increase. The results reveal that experiencing stressful life events in a single social context was not associated with an increased risk of self-injury. However, when stressful life events accumulated across multiple contexts, the risk of self-injury increased (threshold of 2+ contexts from ages 13 to 17, and 3+ contexts at age 20). Similar to the findings for the cumulative count of life events, we found that associations between the number of contexts in which stressful events had occurred and self-injury were stronger and more consistent in females than in males.

### Follow-Up Analyses

The prospective longitudinal design of our study allowed us to investigate a sequence of recent events and self-injury, especially because the data collection process incorporated a timeline that strengthens inferences with respect to the direction of effects (i.e., stressful life events reported for the previous years and self-injury reported for the previous month). Nevertheless, it is possible that adolescents with prior self-injury were at higher risk of exposure to stressful events than those without prior self-injury [e.g., interpersonal stressful events (40)]. Thus, for some of the associations examined here, the self-injury and the event may have had a common cause or the association may be bidirectional in nature.

To control for a potential overlap between life events and prior self-injury, we carried out a set of exploratory follow-up analyses in which we adjusted for self-injury reported in the previous wave when examining associations between life events and later self-injury (i.e., in the model presented in Table 1 and in the overall models with an accumulated number of events, we allowed for auto-regressive paths from self-injury at the previous time-point to the current time-point). This was not possible for predictions of self-injury at age 13 as self-injury assessments prior to age 13 were not available.

Most associations found in the previous models were replicated in these follow-up analyses and remained significant at *p* < 0.05. Only some associations were attenuated. Specifically, the associations between school-related events and self-injury at age 17 (*p* = 0.084) and between family-related events and self-injury at age 15 (*p* = 0.051) were weakened, and the associations between intimate relationship breakups and self-injury at age 17, and between family-related events and self-injury at age 20 were non-significant (*p* = 0.10 and 0.15, respectively). In the threshold model, the association between four or more events and self-injury at age 20 was attenuated (*p* = 0.081).

## Discussion

The results of our study reveal that stressful life events in the contexts of school, peer networks, intimate relationships, and family are all associated with self-injury at some point during adolescence. School- and peer-related life events were consistently associated with self-injury across all ages, but associations were more age-specific for intimate relationship- and family-related life events. An accumulation of two or more stressful life events was strongly associated with an increased risk of self-injury in females from early to late adolescence, but not in males. We discuss the various findings in turn.

### Contexts of Stressful Life Events

School-related life events were associated with self-injury at all ages. The size of this association gradually increased with age, perhaps reflecting the increasing pressures on older adolescents to achieve school and future professional success. Academic failures can be humiliating for adolescents and may cause a reduction in self-esteem, which is a known correlate of self-injury (19, 41). School events may also predict self-injury because repeating a grade or failing an important exam may lower adolescents' social standing among their peers and entail changes in peer groups and schools, which come with additional stressors.

At age 15, when decisions about future vocational schooling or apprenticeships vs. the opportunity to attend academic high school are made in Zurich, associations between school-related life events and self-injury were especially strong in females. It is possible that whole social networks of female adolescents (i.e., groups of friends or close class-mates) are stressed by school-related events in mid-adolescence. This could give rise to social contagion of self-injury (42). In contrast, school-related events were not associated with self-injury in males at age 15. Perhaps males are less stressed by academic tracking or react differently to school-related stressors (e.g., by externalizing problems). Or perhaps they are able to use school changes as an opportunity to renegotiate and improve their social and school standing (43) and to adopt different coping strategies with their new peers in new schools.

Life events involving peer violence were associated with the risk of self-injury across all ages. The major significance of the peer context for adolescent mental health is consistent with prior studies of chronic peer stressors (24, 27) and also with research on self-injurious behaviors (SIB) in non-human primates, which has shown that aggression from peers can be a proximal cause of SIB (44). Among human adolescents, the detrimental effects of peer-related stressful events likely reflect the great importance of peer relationships in adolescents' everyday life and the relevance of these relationships for their well-being (8, 22, 45). Indeed, in our study, peer-related life events were more strongly associated with self-injury than breakups of intimate relationships (although it is also important to consider the different time frames incorporated in the two measures). Notably, the peer events included here involved physical/sexual violence. Violence can have traumatic effects, which may explain why peer events were the strongest correlate of self-injury in our analyses. Indeed, being physically attacked or sexually harassed can elicit a physiological fight-or-flight stress response, which may, in turn, trigger the use of self-injury as a maladaptive coping strategy that reduces physiological arousal and negative affect (3, 4). Although measured as life events here, peer violence could also be associated with chronic forms of victimization, such as bullying. Future research is needed to compare the effects of sporadic physical victimization experiences to those of chronic physical and psychological victimization experiences (e.g., due to exclusion or intimidation by peers).

Stressful life events in the context of intimate relationships showed the weakest and least consistent associations with self-injury: no association in males and a uniquely significant association in females at age 15 only. Indeed, it has been found that adolescent females tend to be more sensitive to interpersonal stressors than males (7). Moreover, adolescent females have been found to focus on dyadic and exclusive relationships and intimacy more than males (46). This emphasis on intimacy, including in (best) friendships, typically increases after early adolescence and could, in part, explain the sex differences in associations between self-injury and intimate relationship events in mid-adolescence. With regard to the age-15-only association, it is possible that break-ups at that age involve a first serious romantic partner, and therefore a novel kind of stressor. The age-specific pattern is also consistent with prior work, which found that chronic romantic stress (e.g., rejection, arguments, having fewer romantic relationships than one's peers) increased the risk of self-injury among girls with advanced pubertal development (21). However, a caveat is that the items used to assess relationship breakup did not distinguish adolescents who were abandoned by a partner or friend from those who decided to break off the relationship themselves. Although both scenarios are likely to be associated with stress, the latter may also entail relief and a sense of self-efficacy. Had we asked about abandonment by a romantic partner only, the associations with self-injury may have been stronger.

The specific vulnerability of females to stressful events in school and intimate relationship contexts at age 15 provides further insights into the potential reasons for the particularly high prevalence of self-injury among females at that age (31). Importantly, our findings provide potential starting points for counteracting this high prevalence.

The sizes of the associations between family-related stressful life events and self-injury decreased from ages 13 to 17, which could reflect adolescents' increased time away from home. Nevertheless, family-related experiences were unique risk factors for self-injury in early and mid-adolescence, which is when self-injury typically first emerges (5). As self-injury is, in many cases, habitualized and therefore recurrent across adolescence (3, 4, 31), these associations between family-related events and early onset of self-injury can be highly relevant for intervention practices. The findings add to prior research, which has revealed that various forms of chronic family adversity, including child maltreatment, relational trauma, lack of support, and hostility, increase the risk of self-injury in adolescents and young adults. These effects have been found to be partially mediated by depressive symptoms, anxiety, and low self-esteem (19, 23, 47). Research has also shown that more sporadic events involving loss and instability in the family increase young people's risk of suicidality (28), which is a well-known correlate of self-injurious behavior (48, 49).

Some research has suggested that family dynamics that promote self-criticism contribute to the risk of self-injury (19, 50). These dynamics could be activated during times of family instability and loss. Family-related life events (e.g., death of a sibling or parental job loss) impose additional stressors on the whole family system, which could precipitate tension, parental expression of negative emotions and criticism, and lower levels of family support, in addition to the distress and grief associated with the events themselves. Such losses and instability within the family could be most detrimental during early adolescence, a time when young people have not yet developed larger support networks outside the home.

### Accumulation of Stressful Life Events

Notably, experiencing multiple stressful events was associated with an increased risk of self-injury, but the threshold for the number of life events needed to trigger self-injury increased from adolescence to early adulthood (from 2+ to 4+ events). This could indicate less vulnerability to multiple stressors as young people come of age (10). Many impulsive behaviors decrease at some point during early adulthood, as the prefrontal cortex matures and self-regulatory and coping capacities increase (51–54); this might also be the case for self-injury. Interestingly, research on adverse childhood experiences and associated health risks in adulthood has also reported that an accumulation of four or more ACE categories can be particularly detrimental (26). However, while ACEs tend to be highly correlated with each other, most of the correlations among stressful life events in the different contexts examined here were modest, indicating an accumulation of independent stressors for any number of reasons.

Alternatively, decreasing associations between cumulative life events and self-injury in early adulthood could indicate that self-injury is replaced by other maladaptive coping strategies (e.g., substance use, other risky behaviors) in the face of major stressful life events. Indeed, although the literature reports that self-injury mostly ceases by adulthood, there is evidence of enduring psychosocial and psychiatric impairment among those previously affected (55). Furthermore, among those who still self-injure in young adulthood, self-injury may have become an entrenched coping mechanism, which is associated with psychiatric disorders [e.g., borderline personality disorder (48)], and does not necessarily require major events in order to be triggered.

An accumulation of stressful life events was consistently associated with self-injury in females. This is consistent with Nock's (3) theory, according to which self-injury is typically a response to the experience of stress. Life events and their accumulation likely evoke over-arousal in adolescent females and place unmanageable demands on them, which are compounded by pressures such as keeping up at school. These unmanageable demands may contribute to feelings of being overwhelmed, which, in turn, triggers self-injury as a maladaptive strategy to alleviate distress.

In contrast, males' risk of self-injury only increased with exposure to four or more stressful events and only at ages 13 and 17. Perhaps most adolescent males turn to more male-typical maladaptive coping behaviors in response to moderate levels of stress, including substance use and delinquent behaviors. In early adolescence (i.e., age 13), they may not yet have the necessary resources to engage in such behaviors. This interpretation is consistent with the pragmatic hypothesis of self-injury emergence (3), according to which young people chose self-injury as a coping strategy because it is easily accessible. For females, self-injury may be a coping mechanism consistent with gender stereotypes and easily accessible means. For mid-adolescent males, self-injury may not conform to male-typical behavior because it is less prevalent among their male peer group [for the sex-specific prevalence of self-injury in the present sample, see (31)], and could, thus, carry an extra cost of stigmatization. Accordingly, adolescent boys may engage in more male-typical mechanisms, including substance use, as soon when such mechanisms become more easily accessible [the legal age for purchasing beer and wine in Switzerland is age 16, and many adolescents initiate use earlier (Quednow et al., under review)].

Nevertheless, some males do engage in self-injury from mid-adolescence to young adulthood, and more research is needed to better understand male-specific triggers of self-injury. It may be worth exploring in more detail some of the contexts examined here, such as the family context in early adolescence and the peer context over the entire period of adolescence, since our analyses show that stressful events in these contexts and at these times are significantly associated with an increased risk of self-injury in males. In addition, researchers may need to look elsewhere for triggers of male self-injury. For example, our previous work on services use suggested that male self-injury could be associated with learning difficulties and concentration and attention problems (31), many of which would not have been captured in the life events categories used here.

### Implications for Practice

In order to protect young people from self-injury, efforts to reduce the number of stressors that adolescents encounter in their daily lives should ideally be combined with efforts to strengthen young people's protective resources.

#### Reducing the Number of Stressors

Young people are inevitably exposed to at least some stressful events during their adolescence (9). Our findings show that especially stressful life events in the contexts of school and peer networks could precipitate self-injury. One important point for prevention and intervention measures to address self-injury is the necessity to reduce peer violence at all stages of adolescence. It is also crucial that policy-makers, when designing school systems and curricula (e.g., in terms of tracking and the timing of transitions and important exams), take into account the fact that increasing school pressures can become toxic and counterproductive and can provoke detrimental responses in youth who are simultaneously facing many other changes in their lives. Such compounding pressures could especially increase distress to an extent that triggers self-injury in adolescent females.

#### Strengthening Protective Factors

Teaching adolescents adaptive coping and social skills (e.g., interactive problem-solving, help seeking, strategies for emotion-regulation) is vital to prevent the use of self-injury and the potential long-term psychiatric impairment associated with this behavior. Mental health care providers should also work with adolescents to improve their social support networks (56). In addition, services may need to be tailored to the specific challenges that adolescents face (e.g., school-based support services to address school-related problems) but should also include a comprehensive focus on potential stressors in other contexts of adolescent everyday life. Furthermore, for families of young adolescents, it may be important to counteract dynamics that foster self-criticism in the face of stress (50).

### Limitations

Our study is not without limitations. First, it is not ideal to assess self-injury with one survey item only and a limited list of example behaviors, although the use of single-item measures is common in self-injury research (2). The exclusion of some male-typical self-injurious behaviors (e.g., punching a fist into a wall) may have led to underestimations of the associations between stressful life events and self-injury among males.

Second, our findings may not be generalizable to other school systems. The educational system of the canton of Zurich entails several major educational transitions during the adolescent period, which may offer adolescents opportunities to overcome certain prior disadvantages [e.g., children who were previously rejected by their peers in class could encounter new opportunities for building more positive relationships with their new classmates at a new school (43)]. However, these transitions can also impose additional stressors on young people at a time when they are particularly vulnerable (57, 58). Investigations of community-representative samples from other regions are needed to explore whether the age-specific patterns we observed can be replicated. However, adolescents in many other Western countries face similar major transitions (e.g., the transition to middle school and then high school in the United States) and educational pressures, which could explain why self-injury is a major problem in many of these countries (17).

Third, the list of stressful life events used in our study is reasonably comprehensive but, necessarily, selective. Therefore, we may have missed effects of other important events (e.g., violence by an intimate partner or family member). Including additional stressful events could also alter our conclusions with regard to sex differences (for example, in case that we missed events that increase stress levels more among males than females). Notwithstanding this limitation, we were able to show that, at least at some points across adolescence, events in each of the four contexts had unique associations with the risk of self-injury. Future analyses should consider the role of events that are bound to particular ages or life phases (e.g., major life-course transitions pending in early adulthood, such as labor market entry, parenthood, or marriage, or events involving intimate partner violence, which may become more prevalent in and after mid-adolescence when enduring partnerships become normative).

Fourth, although our prospective longitudinal study design and the timeline incorporated into the data collection processes strengthen inferences with respect to the direction of effects, we cannot draw ultimate conclusions with regard to causality. The attenuation of some associations between stressful events and self-injury in the models with autoregressive effects may indicate, in some cases, common underlying causes or reciprocal effects between particular events and self-injury (40). For example, experiences of loss and separation in a family and a child's self-injury in mid-adolescence could result from the same (earlier) family disruption (e.g., illness or conflicts and, as a consequence, increased stress levels and limited opportunities for learning more adaptive emotion regulation strategies). Ideally, future research would include both, adverse childhood experiences and acute stressful events in adolescence, to compare their relevance in the emergence of adolescent self-injury. With regard to the associations between self-injury and intimate relationship breakups, emotional instability could be a common cause, especially in a small subset of adolescents who are developing borderline personality disorder, which is often characterized by both frequent relationship instability and self-injury (59). Nevertheless, most associations remained significant in the analyses with auto-regressive effects, and we can be somewhat confident about the sequence of events (i.e., self-injury follows stressful events in different social contexts).

Fifth, assessing life events that occurred during the previous one to three years and self-injury during the previous month could, in some cases, mean that the time elapsed between an event and self-injury spans almost one, two, or three years. This long period may have deflated associations in our analyses.

Finally, it is possible that using the same data for multiple hypotheses testing may have caused alpha inflation. However, our major findings were typically significant at *p* < 0.005.

## Conclusions

Adolescent self-injury is a complex phenomenon. Our findings suggest that various pathways could lead to overwhelming distress that triggers self-injury (3). This includes exposure to life events that are particularly detrimental (e.g., being the victim of violence committed by a peer) but also an accumulation of stressful life events that become unmanageable, irrespective of the context in which the events occur. Future research is needed on age- and sex-specific associations between self-injury and stressful events in different contexts of adolescent life, as well as the overall stress burden that young people face, to better understand when and why a stress-response will manifest itself in self-injury. Such research might also benefit from assessing biological stress levels to pinpoint who will engage in self-injury.

## Data Availability Statement

The datasets generated for this study will not be made publicly available due to sensitive personal information and the potentially socially stigmatizing nature of self-injury. Requests from scientists for re-analysis can be directed to the first author.

## Ethics Statement

The studies involving human participants were reviewed and approved by the responsible ethics committee at the Faculty of Arts and Social Sciences, University of Zurich. Adolescents provided written informed consent at each wave; parents of those aged 15 and younger could choose not to have their child participate in the study.

## Author Contributions

AS and LS conceived of and drafted the manuscript. ME and DR designed the survey and conducted the data acquisition. DR, ME, AS, and LB prepared the data for analyses. AS took the lead in analyzing the data, with contributions from LB. AS, LB, DR, ME, and LS critically revised the manuscript and contributed important intellectual content. All authors approved the manuscript.

## Conflict of Interest

The authors declare that the research was conducted in the absence of any commercial or financial relationships that could be construed as a potential conflict of interest.
