# Supplementary material for: Stressful Life Events in Different Social Contexts Are Associated With Self-Injury From Early Adolescence to Early Adulthood
Source: Front Psychiatry. 2020 Oct 27;11:487200. doi: 10.3389/fpsyt.2020.487200 (PMC7653177; doi:10.3389/fpsyt.2020.487200)
Supplement: Supplementary file 1 [file Data_Sheet_1.PDF]

Supplementary Material

1 Stressful Life Events Among Youth with and Without Self-Injury

Frequencies: Tables S1 and S2 show the frequency with which events occurred in the various contexts (Table S1) among youth with and without self-injury and the frequency with which particular types of events (Table S2) occurred, which were categorized into contexts for the main analyses.

Mean scores: Overall, adolescents with self-injury experienced a higher average number of stressful life events than their peers without self-injury at all assessments (2.20 versus 1.36, $p < 0.001$; 1.93 versus 1.25, $p < 0.001$; 1.74 versus 1.14, $p < 0.001$; 2.78 versus 1.88, $p < 0.001$ at ages 13, 15, 17, and 20, respectively).

Among males, group differences between those with and without self-injury were observed sporadically (2.16 versus 1.32, $p < 0.001$; 1.32 versus 1.11, $p = 0.17$; 1.54 versus 0.98, $p < 0.001$; 1.97 versus 1.56, $p = 0.11$ at ages 13, 15, 17, and 20, respectively). Among females there were significant group differences between those with and without self-injury across the entire period from early adolescence to early adulthood (2.24 versus 1.41, $p < 0.001$; 2.22 versus 1.42, $p < 0.001$; 1.82 versus 1.33, $p < 0.001$; 3.17 versus 2.22, $p < 0.001$ at ages 13, 15, 17, and 20).

Table S1. Prevalence of any stressful life events in the four social contexts among youth with and without self-injury, overall and sex-specific

	**School**			**Peers**			**Intimate relationships**			**Family**		
	with self-injury % *n/N*	without self-injury % *n/N*	*p* for group diff.	with self-injury % *n/N*	without self-injury % *n/N*	*p* for group diff.	with self-injury % *n/N*	without self-injury % *n/N*	*p* for group diff.	with self-injury % *n/N*	without self-injury % *n/N*	*p* for group diff.
**Overall**												
Age 13	38.4 *68/177*	28.4 *336/1185*	0.006	52.0 *92/177*	26.2 *310/1184*	< 0.001	50.8 *90/177*	42.1 *498/1184*	0.028	49.2 *87/177*	29.1 *345/1185*	< 0.001
Age 15	28.5 *47/165*	18.3 *234/1278*	0.002	44.8 *74/165*	25.6 *327/1276*	< 0.001	52.1 *86/165*	39.8 *508/1277*	0.002	43.6 *72/165*	30.7 *392/1278*	< 0.001
Age 17	23.0 *29/126*	13.5 *158/1174*	0.004	45.2 *57/126*	21.7 *255/1177*	< 0.001	49.2 *62/126*	36.7 *430/1171*	0.006	34.1 *43/126*	33.4 *392/1174*	0.868
Age 20	34.4 *33/96*	18.9 *205/1084*	< 0.001	52.1 *50/96*	29.1 *315/1084*	< 0.001	61.5 *59/96*	55.4 *601/1084*	0.255	55.2 *53/96*	42.7 *463/1084*	0.018
**Males**												
Age 13	45.3 *39/86*	30.6 *188/615*	0.006	43.0 *37/86*	22.6 *139/615*	< 0.001	51.2 *44/86*	41.3 *254/615*	0.083	48.8 *42/86*	28.1 *173/615*	< 0.001
Age 15	18.9 *10/53*	19.7 *137/694*	0.878	28.3 *15/53*	16.6 *115/692*	0.031	34.0 *18/53*	36.7 *255/694*	0.685	43.4 *23/53*	29.0 *201/694*	0.027
Age 17	29.7 *11/37*	13.8 *85/617*	0.008	27.0 *10/37*	11.8 *73/619*	0.007	45.9 *17/37*	34.1 *210/616*	0.141	27.0 *10/37*	31.3 *193/617*	0.587
Age 20	29.0 *9/31*	19.3 *106/550*	0.185	38.7 *12/31*	16.6 *91/549*	0.002	48.4 *15/31*	51.8 *285/550*	0.710	45.2 *14/31*	43.3 *238/550*	0.836
**Females**												
Age 13	31.9 *29/91*	26.0 *148/570*	0.238	60.4 *55/91*	30.1 *171/569*	< 0.001	50.5 *46/91*	42.9 *244/569*	0.171	49.5 *45/91*	30.2 *172/570*	< 0.001
Age 15	33.0 *37/112*	16.6 *97/584*	< 0.001	52.7 *59/112*	36.3 *212/584*	0.001	60.7 *68/112*	43.4 *253/583*	< 0.001	43.8 *49/112*	32.7 *191/584*	0.024
Age 17	20.2 *18/89*	13.1 *73/557*	0.073	52.8 *47/89*	32.6 *182/558*	< 0.001	50.6 *45/89*	39.6 *220/555*	0.052	37.1 *33/89*	35.7 *199/557*	0.805
Age 20	36.9 *24/65*	18.5 *99/534*	< 0.001	58.5 *38/65*	41.9 *224/534*	0.011	67.7 *44/65*	59.2 *316/534*	0.186	60.0 *39/65*	42.1 *225/534*	0.006

Table S2. Prevalence of particular types of stressful life events among youth with and without self-injury

	**Age 13**			**Age 15**			**Age 17**			**Age 20**		
Context and type of event	with self-injury %<br>*n/N*	without self-injury %<br>*n/N*	*p* for group diff.	with self-injury %<br>*n/N*	without self-injury %<br>*n/N*	*p* for group diff.	with self-injury %<br>*n/N*	without self-injury %<br>*n/N*	*p* for group diff.	with self-injury %<br>*n/N*	without self-injury %<br>*n/N*	*p* for group diff.
**School**												
Grade retention	10.2<br>*18/176*	3.7<br>*44/1183*	< 0.001	6.7<br>*11/164*	3.5<br>*45/1273*	0.048	12.7<br>*16/126*	4.9<br>*57/1171*	< 0.001	17.7<br>*17/96*	6.5<br>*71/1084*	< 0.001
Exam failure	29.5<br>*52/176*	25.3<br>*299/1183*	0.227	24.8<br>*41/165*	15.6<br>*199/1276*	0.003	14.4<br>*18/125*	10.0<br>*117/1172*	0.124	27.1<br>*26/96*	16.9<br>*183/1084*	0.012
**Peers**												
Violent assault	29.9<br>*53/177*	12.5<br>*148/1183*	< 0.001	14.5<br>*24/165*	10.0<br>*127/1274*	0.071	8.7<br>*11/126*	5.2<br>*61/1177*	0.098	14.6<br>*14/96*	5.9<br>*64/1084*	0.001
Sexual victimization	36.7<br>*65/177*	16.3<br>*193/1184*	< 0.001	38.2<br>*63/165*	18.7<br>*238/1276*	< 0.001	39.7<br>*50/126*	17.7<br>*208/1177*	< 0.001	41.7<br>*40/96*	25.4<br>*275/1084*	< 0.001
**Intimate Relationships**												
Best friendship breakup	19.2<br>*34/177*	14.2<br>*168/1181*	0.082	23.0<br>*38/165*	16.5<br>*211/1276*	0.038	24.6<br>*31/126*	15.5<br>*181/1170*	0.008	41.7<br>*40/96*	24.9<br>*270/1084*	< 0.001
Romantic relationship breakup	43.8<br>*77/176*	34.1<br>*402/1180*	0.012	40.0<br>*66/165*	30.0<br>*382/1272*	0.009	38.9<br>*49/126*	27.5<br>*321/1166*	0.007	50.0<br>*48/96*	44.8<br>*485/1083*	0.325
**Family**												
Loss	4.0<br>*7/177*	1.6<br>*19/1185*	0.033	≤ 3.1<br>*≤ 5/165*	1.4<br>*18/1277*	0.317	≤ 4.0<br>*≤ 5/126*	1.4<br>*16/1174*	0.838	≤ 5.2<br>*≤ 5/96*	2.4<br>*26/1084*	0.291
Instability	47.5<br>*84/177*	28.5<br>*338/1185*	< 0.001	43.6<br>*72/165*	29.7<br>*380/1278*	< 0.001	33.3<br>*42/126*	32.9<br>*386/1174*	0.918	54.2<br>*52/96*	42.2<br>*457/1084*	0.023

2 Associations Between Life Events in Different Social Contexts

Table S3. Associations between life events in the four contexts at age 13: Odds ratios (95 % CI) from bivariate binary logistic regressions

	School	Peers	Intimate Rel.	Family
School	--			
Peers	1.31 (1.02—1.68)	--		
Intimate Relationships	1.28 (1.02—1.62)	2.99 (2.35—3.80)	--	
Family	1.59 (1.25—2.03)	1.63 (1.28—2.09)	1.42 (1.13—1.78)	--

Table S4. Associations between life events in the four contexts at age 15: Odds ratios (95 % CI) from bivariate binary logistic regressions

	School	Peers	Intimate Rel.	Family
School	--			
Peers	1.57 (1.19—2.08)	--		
Intimate Relationships	1.23 (0.94—1.59)	2.42 (1.91—3.06)	--	
Family	1.52 (1.16—1.99)	1.41 (1.11—1.80)	1.37 (1.10—1.72)	--

Table S5. Associations between life events in the four contexts at age 17: Odds ratios (95 % CI) from bivariate binary logistic regressions

	School	Peers	Intimate Rel.	Family
School	--			
Peers	1.68 (1.20—2.35)	--		
Intimate Relationships	1.40 (1.03—1.92)	2.14 (1.65—2.77)	--	
Family	1.19 (0.87—1.65)	1.21 (0.93—1.59)	1.06 (0.84—1.34)	--

Table S6. Associations between life events in the four contexts at age 20: Odds ratios (95 % CI) from bivariate binary logistic regressions

	School	Peers	Intimate Rel.	Family
School	--			
Peers	1.35 (1.00—1.81)	--		
Intimate Relationships	1.32 (0.99—1.77)	2.52 (1.94—3.29)	--	
Family	1.53 (1.15—2.03)	1.28 (1.00—1.64)	1.29 (1.03—1.63)	--

3 Sex-Specific Models Including Stressful Life Events in the four Social Contexts

Table S7. Associations between self-injury between age 13 and 20 (dependent variables) and life events: multivariate logistic regressions with multiple imputation, males (n = 767)

				95% CI	
	**logit coeff. *b***	***p***	**OR**	**lower**	**upper**
**Self-injury age 13**					
High parental education	0.15	0.626	1.16	0.63	2.13
Parents divorced by child age 11	0.65	0.047	1.92	1.01	3.63
Migration background	0.18	0.499	1.19	0.72	1.99
Context of event					
School	0.50	0.042	1.65	1.02	2.69
Peers	0.76	0.002	2.15	1.33	3.47
Intimate relationships	0.08	0.744	1.08	0.68	1.73
Family	0.62	0.012	1.86	1.15	3.03
**Self-injury age 15**					
High parental education	-0.96	0.041	0.38	0.15	0.96
Parents divorced by child age 11	-0.30	0.477	0.74	0.32	1.70
Child educational level (high)[a]	-0.11	0.840	0.90	0.32	2.52
Migration background	0.02	0.936	1.02	0.58	1.82
Context of event					
School	-0.17	0.630	0.85	0.43	1.68
Peers	0.71	0.027	2.04	1.08	3.83
Intimate relationships	-0.23	0.468	0.80	0.43	1.47
Family	0.64	0.030	1.90	1.07	3.40
**Self-injury age 17**					
High parental education	-1.66	0.009	0.19	0.05	0.66
Parents divorced by child age 11	-0.05	0.922	0.95	0.36	2.51
Child educational level (high)[a]	0.54	0.206	1.71	0.74	3.94
Migration background	-0.36	0.358	0.70	0.33	1.50
Context of event					
School	0.91	0.014	2.49	1.20	5.19
Peers	1.01	0.011	2.75	1.26	6.00
Intimate relationships	0.38	0.287	1.46	0.73	2.93
Family	-0.26	0.499	0.77	0.36	1.64
**Self-injury age 20**					
High parental education	-0.46	0.376	0.63	0.23	1.74
Parents divorced by child age 11	-0.05	0.927	0.96	0.37	2.49
Child educational level (high)[a]	-1.12	0.077	0.33	0.10	1.13
Migration background	0.47	0.235	1.60	0.74	3.46
Context of event					
School	0.51	0.241	1.66	0.71	3.87
Peers	1.23	0.004	3.41	1.49	7.81
Intimate relationships	-0.32	0.443	0.73	0.33	1.64
Family	-0.22	0.548	0.80	0.39	1.66

[a] Education level at the previous assessment

Table S8. Associations between self-injury between age 13 and 20 (dependent variables) and life events: multivariate logistic regressions with multiple imputation, females (n = 715)

				95% CI	
	**logit coeff. *b***	***p***	**OR**	**lower**	**upper**
**Self-injury age 13**					
High parental education	-0.68	0.053	0.51	0.25	1.01
Parents divorced by child age 11	0.12	0.732	1.13	0.57	2.22
Migration background	-0.27	0.255	0.76	0.48	1.22
Context of event					
School	0.30	0.254	1.34	0.81	2.23
Peers	1.22	< 0.001	3.37	2.07	5.50
Intimate relationships	-0.16	0.531	0.86	0.52	1.40
Family	0.66	0.007	1.93	1.20	3.12
**Self-injury age 15**					
High parental education	-0.35	0.216	0.71	0.41	1.23
Parents divorced by child age 11	0.40	0.178	1.49	0.83	2.66
Child education level (high)[a]	-0.01	0.972	0.99	0.54	1.80
Migration background	-0.45	0.047	0.64	0.41	0.99
Context of event					
School	0.80	0.001	2.22	1.37	3.62
Peers	0.42	0.060	1.52	0.98	2.36
Intimate rel.	0.52	0.017	1.69	1.10	2.60
Family	0.38	0.084	1.46	0.95	2.24
**Self-injury age 17**					
High parental education	0.21	0.480	1.24	0.69	2.22
Parents divorced by child age 11	0.22	0.438	1.24	0.72	2.15
Child education level (high)[a]	-0.18	0.553	0.84	0.47	1.50
Migration background	-0.30	0.242	0.74	0.44	1.23
Context of event					
School	0.45	0.151	1.57	0.85	2.89
Peers	0.74	0.003	2.09	1.29	3.38
Intimate relationships	0.33	0.168	1.40	0.87	2.24
Family	0.06	0.795	1.06	0.66	1.71
**Self-injury age 20**					
High parental education	0.24	0.523	1.27	0.61	2.61
Parents divorced by child age 11	0.43	0.192	1.53	0.81	2.91
Child education level (high)[a]	-0.10	0.776	0.91	0.47	1.75
Migration background	-0.38	0.193	0.69	0.39	1.21
Context of event					
School	1.01	0.001	2.74	1.53	4.92
Peers	0.58	0.037	1.79	1.04	3.10
Intimate relationships	0.13	0.663	1.13	0.64	2.00
Family	0.71	0.013	2.03	1.16	3.55

[a] Education level at the previous assessment

4 Distribution of Cumulative Stressful Life Events Variable

Table S9. Group sizes (n) of youth with particular numbers of stressful life events between age 13 and 20

Number of stressful life events	Overall	Male	Female
Age 13	*1362*	*701*	*661*
1	453	246	207
2	309	151	158
3	161	68	93
4+	102	55	47
Age 15	*1443*	*747*	*696*
1	473	261	212
2	313	144	169
3	162	60	102
4+	78	26	52
Age 17	*1305*	*658*	*647*
1	462	236	226
2	281	122	159
3	123	45	78
4+	40	11	29
Age 20	*1180*	*581*	*599*
1	313	183	130
2	249	135	114
3	190	69	121
4+	210	58	152

5 Cumulative Life Events Across Contexts and Self-Injury

In these supplemental analyses, we examined how the number of contexts in which life events are occurring is associated with self-injury. The rationale was that overwhelming stressors may trigger self-injury, and that such overwhelming stressors may arise when life events pervade multiple contexts of adolescents' everyday lives.

Measure. We used a sum score indicating the number of contexts in which stressful events occurred.

Analytic Approach. We specified models in which the number of contexts in which stressful events occurred was included as categorical variables. Zero contexts served as the reference category.

Overall sample. Experiencing stressful events in two contexts was associated with an increased risk of self-injury from early to late adolescence (OR [age 13] = 2.15, 95% CI = 1.30–3.57; OR [age 15] = 1.91, 95% CI = 1.17–3.13; OR [age 17] = 2.28, 95% CI = 1.27–4.07) but not at age 20. Youth who reported stressful events in the majority of social contexts (i.e., 3+) had a significantly higher risk of self-injury from early adolescence until early adulthood. Although the effect size decreased over time, it remained large until age 20 (OR [age 13] = 3.89, 95% CI = 2.37–6.40; OR [age 15] = 3.31, 95% CI = 1.97–5.55; OR [age 17] = 3.67, 95% CI = 1.84–7.29; OR [age 20] = 2.77, 95% CI = 1.54–4.98).

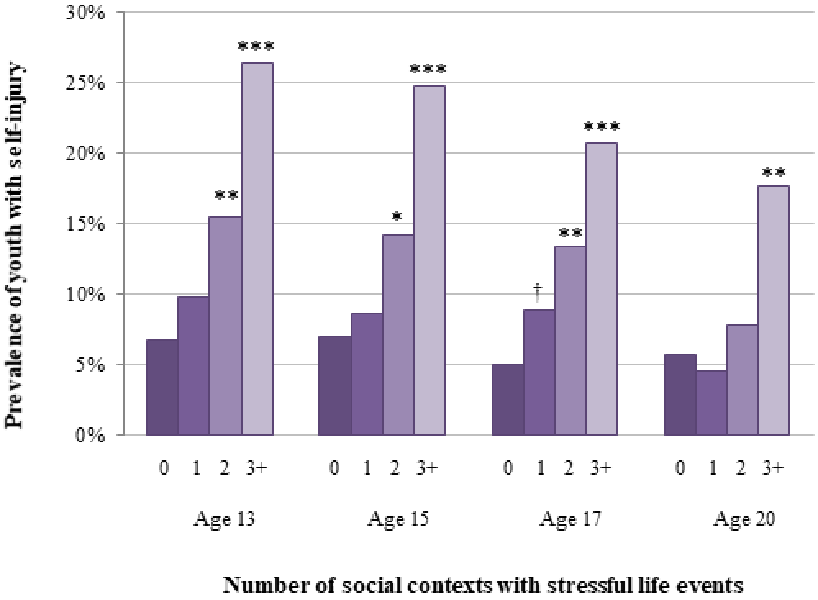

Figure S1. Overall prevalence of youth with self-injury among groups who experienced stressful events in zero to three or more social contexts from ages 13 to 20. Asterisks represent *p* showing the contrast between particular numbers of contexts versus zero contexts/events (reference) from a model that adjusted for sex, parental educational background, adolescent educational level at previous assessment, parental divorce by age 11, and migration background. †$p < 0.10$; *$p < 0.05$, **$p < 0.01$, ***$p < 0.001$

By sex. Males' risk of self-injury significantly increased with exposure to stressful events in three or more contexts at age 13 (OR = 3.94, 95% CI = 1.95–7.99). There was also a significant difference in prevalence of self-injury between those with stressful events in two and those with events in zero contexts/events at age 17 (OR = 2.49, 95% CI = 1.06–5.88). The non-significant contrast between those with events in three or more contexts versus zero contexts/events could be due to a relatively small group size (n = 32). In contrast, the pattern for females was similar to the overall pattern: stressful events in two contexts increased the risk of self-injury across the entire period from early to late adolescence (OR [age 13] = 3.21, 95% CI = 1.59–6.46; OR [age 15] = 2.63, 95% CI = 1.30–5.31; OR [age 17] = 2.06, 95% CI = 1.01–4.22), but not in early adulthood. Experiencing events in three or more contexts had an even larger effect size; this effect remained significant until age 20 (OR [age 13] = 4.49, 95% CI = 2.14–9.42; OR [age 15] = 5.38, 95% CI = 2.62–11.03; OR [age 17] = 3.57, 95% CI = 1.59–8.05; OR [age 20] = 3.59, 95% CI = 1.49–8.65).

Taken together, the analyses of the overall sample and females suggest that experiencing life events in two or more contexts is associated with self-injury throughout adolescence. In young adulthood, when stress reactivity may have decreased and the variety of coping strategies may have increased, experiencing life events in three or more contexts was associated with self-injury. Notably, the number of contexts in which life events occurred predicted male self-injury at ages 13 and 17 only, but not at other ages.

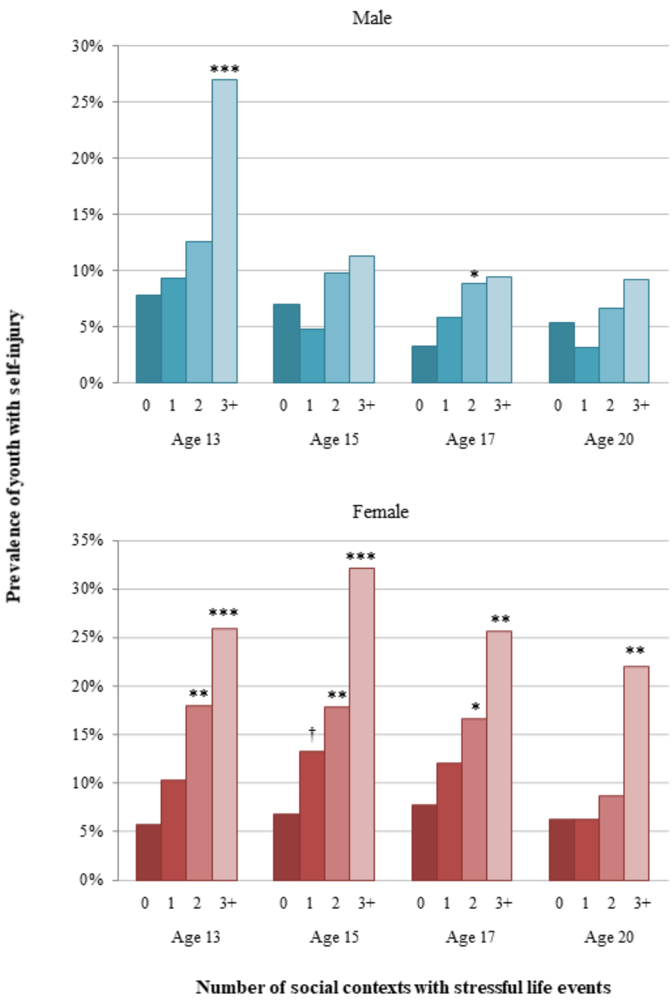

Figure S2. Sex-specific proportion of youth with self-injury among groups with stressful events in zero to three or more social contexts from ages 13 to 20. Asterisks represent *p* showing the contrast between particular number of contexts versus zero contexts/events (reference) from models that adjusted for parental educational background, adolescent educational level at previous assessment, parental divorce by age 11, and migration background. †$p < 0.10$; *$p < 0.05$, **$p < 0.01$, ***$p < 0.001$
